# The Potential of *Magnolia* spp. in the Production of Alternative Pest Control Substances

**DOI:** 10.3390/molecules28124681

**Published:** 2023-06-09

**Authors:** Juana Valeria Hernandez-Rocha, Suria Gisela Vásquez-Morales

**Affiliations:** Department of Biology, Division of Natural and Exact Sciences, University of Guanajuato, Guanajuato 36050, Mexico

**Keywords:** magnoliaceae, bioprospecting, conservation, botanical pesticides, pollyfollicle, seed, sarcotesta

## Abstract

The irrational use of synthetic pesticides in agriculture has had negative impacts on ecosystems and contributed to environmental pollution. Botanical pesticides offer a clean biotechnological alternative to meet the agricultural challenges posed by pests and arthropods. This article proposes the use of fruit structures (fruit, peel, seed, and sarcotesta) of several *Magnolia* species as biopesticides. The potential of extracts, essential oils, and secondary metabolites of these structures for pest control is described. From 11 *Magnolia* species, 277 natural compounds were obtained, 68.7% of which were terpenoids, phenolic compounds, and alkaloids. Finally, the importance of a correct management of *Magnolia* species to ensure their sustainable use and conservation is stressed.

## 1. Introduction

One of the main problems faced in the production of fruits and vegetables around the world is pest control [1]. The Food and Agriculture Organization of the United Nations (FAO) reports that 40% of total world agricultural production is lost to pests [2], mostly of the Hexapoda class (insects) and the orders, Coleoptera, Diptera, Hemiptera, Homoptera, Hymenoptera, Lepidoptera, Orthoptera, and Thysanoptera [3,4].

Among the many crop protection methods known today, chemical control (pesticides) remains the most widely used one [5]. Applying synthetic pesticides to crops is an effective way to reduce the production losses. However, their toxicity poses serious risks. Pesticides are chemical compounds or combinations of them used to repel, destroy, and control pests [6]. Generally, they are characterized as highly effective, wide-spectrum chemical substances, but the majority of them are also highly toxic and contaminates the ecosystem. According to the target organism, pesticides are classified as insecticides, molluscicides, acaricides, fungicides, bactericides, or others [7]. In the case of insecticides, the most environmentally harmful groups are organochlorines, organophosphates, carbamates, and pyrethroids [8].

The Integral Pest Control promoted by the FAO consists of monitoring, regulating, and controlling pests via sound methods compatible with the natural environment, while reducing the use of toxic pesticides that affect the life of non-target organisms, the environment, and natural resources [9]. An alternative to synthetic pesticides is the use of pesticides of botanical origin, whose action may be comparatively slower but are safer and environmentally friendly [8,9,10]. They are also known as natural pesticides and biopesticides and may come in the form of botanical extracts, essential oils, and natural compounds [11,12].

Botanical pesticides offer several advantages over synthetic pesticides. In fact, they are as effective or even superior to their counterparts [13]. They pose minimal risks to other organisms, such as mammals (including humans), birds, reptiles, and plants. As they are of natural origin, their degree of persistence and accumulation in the environment are very low. They are photosensitive biodegradable molecules that are easily decomposed by solar rays and the action of microorganisms [14]. For example, it has been demonstrated that azadirachtin remains in the soil and cultivars for 24 to 48 h [15], while pyrethrin persists for three to five hours after application, which reduces its potential impact on natural resources (water, soil, and air) and constitutes a beneficial attribute for environmental conservation [14]. Another important characteristic of botanical insecticides is their multispecific action, which makes it more difficult for pests to develop resistance to the compound, as opposed to chemical pesticides, which regularly target a specific molecule [16]. For example, flavanones were evaluated in terms of *Mycobacterium tuberculosis* viability to act against protein kinase G (PknG) as a new promising drug target [17], and fukugetin, a natural flavone as an inhibitor of human tissue kallikreins [18].

In this article, the biopesticide potential of botanical extracts and essential oils obtained from the fruit (seeded and whole), seeds, and sarcotesta of several *Magnolia* species is analyzed in detail. To understand their effectiveness in pest control, the biological activity of their secondary metabolites is described. The bibliographic review conducted here confirms the benefits of using *Magnolia* species as natural biopesticides in agroecosystems and stresses the need to promote their conservation and further the study of this taxonomic group from an ecological perspective.

## 2. The Magnoliaceae Family

**Figure 1:**
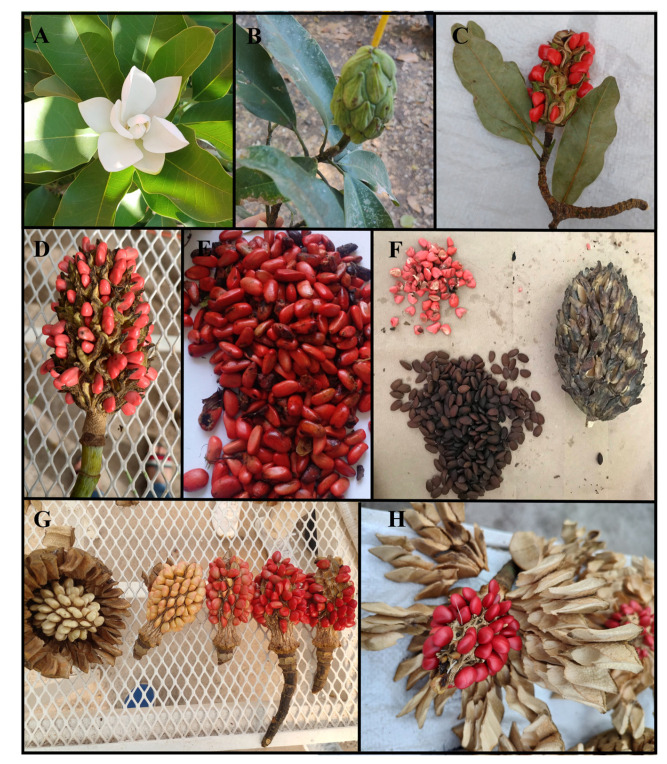
Morphological characteristics of *Magnolia*. (**A**) flower of *M. pugana*. (**B**) Immature fruit of *M. pugana*, mature fruit with exposed seeds of (**C**) *M. pugana* and (**D**) *M. vovidesii*. (**E**) Seeds of *M. vovidesii*. (**F**) Sarcotesta (aril) seed without sarcotesta and seedless fruit of *M. vovidesii*. (**G**) Ripening process of fruit with seeds of *M. perezfarrerae*. (**H**) Mature fruit with exposed seeds of *M. perezfarrerae*. Photo credit: Suria Vásquez.

The Magnoliaceae family is among the most primitive living flower plant families. Fossil registries date from the Cretacic period (135–100 million years ago), when dinosaurs were still alive [19,20]. The family is divided into *Liriodendron* and *Magnolia*, the latter genus being the largest one, with a total of 312 species [21]. Most of the species identified (~80%) are distributed in temperate and tropical climate zones in south-east Asia, and a smaller number (~20%) are found in the American continent [22]. *Magnolia* species are easily identified by their morphological characteristics. They are arboreous or shrubby plants with deciduous or evergreen foliage [23]. The flowers are large and solitary, with a perianth of two or more spirals of free tepals (petaloids), many stamens, anthers with two loculaments and carpels arranged in spirals. The fruits are polyfollicles made of joint or separated carpels and they may be dehiscent, circumscissile, or indehiscent, and the seeds are long and wrapped in a crimson red sarcotesta that can be removed from the endocarp [22]. The sarcotesta is also called aril, an edible pulp that covers the seed (Figure 1) [24]. Interestingly, the seeds specifically depend on spreading birds that can detect their red color [25,26].

## 3. *Magnolia* in Traditional Medicine

Some *Magnolia* species have been used in traditional medicine for their therapeutic and pharmacological properties [27]. *Houpu*, a traditional Chinese remedy made with the bark of *M. officinalis*, has been used for millennia to treat “energy (*qi*) stagnation”, the cause of asthma and digestive afflictions, as well as to prevent stress, anxiety, and depression [28]. It has been found that the two main active components of *houpu* are the lignans, magnolol and honokiol [29]. Likewise, in traditional medicine in India, root bark extracts of *M. champaca* have been used to treat tumors, constipation, swelling, amenorrhea, and dysmenorrhea, and its flowers are used to treat chronic gastritis, fever, cough, bronchitis, and heart weakness [30]. In traditional medicine in North America, *Magnolia* has been used to treat several illnesses. The ethnomedical data describe that the tonic obtained from the bark of the root and stem of *M. virginiana* is a remedy for autumn fever, fever paroxysms, and rheumatism [31]. Native communities use an extract of *M. grandiflora* seeds, whose sedative and hypnotic effects help to control sleep and body temperature. This extract has also antispasmodic and anti-inflammatory properties and can eliminate the immunoresistance associated with breast and prostate cancers. It can also be used to treat convulsions and fight microbial infections [32]. In Mexico, infusions of flowers and leaves of *M. yajlachhi* have been used in traditional Zapotec medicine for several purposes, including the strengthening of heart rhythms, invigorating the blood, and the clearing eyes, in addition the aroma of the flowers is used to treat asthma [33]. The decoction of leaves and bark of *M. dealbata* is used as a tranquilizer and anticonvulsant in cases of epilepsy [34].

## 4. *Magnolia* and Sustainable Agriculture

The growing interest in the use of botanical pesticides in agriculture today constitutes a favorable scenario for the application of natural products (botanical extracts, essential oils, and others) derived from *Magnolia* plants. Several reports highlight the biocidal properties of these compounds on insects that afflict plants of commercial value [35,36]. For example, the raw extracts and essential oils from fruits, seeds, and sarcotesta of *Magnolia* spp. have been successfully used against insect infestations [37,38,39], and a large variety of phytochemicals with possible insecticidal properties have been proposed. Sarker and Maruyama [22] and Song and Fischer [40] have documented that some *Magnolia* spp. are rich in lignans, neolignans, alkaloids, flavonoids, and terpenoids, with different biological uses as insecticides, deterrents, repellents, and anti-nutrients [22,23,24,25,26,27,28,29,30,31,32,33,34,35,36,37,38,39,40]. Therefore, the use of natural products derived from *Magnolia* spp. is a good strategy in integral pest management and can help mitigate environmental deterioration and the accumulation of toxic residues derived from the application of synthetic insecticides [41].

## 5. Botanical Extracts and Essential Oils with Insecticidal Properties

The biocidal potential of natural products derived from native plants to combat different pest insects has called the attention of the scientific community [42]. Though the importance of knowing the properties (chemical composition, biomolecules such as proteins, and genes) of the species that make up the local and endemic flora has become increasingly evident, not all the taxonomic groups of the *Magnolia* genus have been sufficiently explored [43]. For example, while *M. officinalis*, an endemic species from China, has been widely studied, the biocidal capabilities of *M. fragarigynandria*, *M. mayae*, *M. narinensis,* and *M. rzedowskiana* remain scarcely known [21,22,23,24,25,26,27,28,29,30,31,32,33,34,35,36,37,38,39,40,41,42,43,44]. Moreover, phytochemical studies of the vegetative structures of *Magnolia* spp. have rarely paid attention to leaf, bark, flower, fruit, seed, and sarcotesta in equal proportions.

The insecticidal properties of *Magnolia* have been confirmed in a study by Kelm et al., [45], in which extracts of hexane and methanol from fruits of *M. salicifolia*, a species endemic to Japan, were given to mosquitoes. The results indicate that both of these extracts had a significant biocide potential (250 ppm in 24 h) on *Ae. aegypti* at the fourth larval stage [45]. Similarly, the insecticidal activity of essential oils of the mature and immature leaves, flowers, and fruits of *M. grandiflora*, a species endemic to the United States, were found to have the worst toxic effects (49.4 and 48.9 ppm) on *Ae. aegypti* larvae. Finally, an essential oil obtained from seeds showed a strong repellent effect (0.89) on adult mosquitoes [46].

Wang et al., [47] run toxicity tests of a substance obtained via the hydro-distillation of seeds of *M. denudata*, a species endemic to China, on larvae of *Culex pipens pallens*, *Ae. aegypti, Ae. albopictus*, and *Anopheles sinensis* and obtained insecticidal bioactivity values of 19.6, 19.3, 21.4, and 24.84 mg/L, respectively [47]. Recently, it was reported that the essential oil of *M. grandiflora* seeds is highly effective against imported hybrid fire ants (*Solenopsis invicta*) [48]. Vásquez-Morales et al., [38] on the other hand, reported that the ethanolic extracts of sarcotesta and seed of *Magnolia schiedeana*, a species endemic to Mexico, have a potential in the development of useful bioinsecticides in the control of adult specimens of *Anastrepha ludens*, the Mexican fruit fly. In the study, ethanolic extracts of leaves, flowers, bark, empty polyfollicles, seeds, and sarcotesta were evaluated, and it was found that only the seed and sarcotesta extracts had significant levels of insecticidal effectiveness (59.3 and 64.7%) against flies [38]. On the same insect, the ethanolic extracts of leaves, flowers, bark, seeds, and sarcotesta of *M. dealbata* (currently *M. vovidesii*, a species endemic to Mexico) were evaluated. It was reported that the ethanolic extracts of sarcotesta showed the highest insecticidal activity level (96%) against *A. ludens* adults [37]. Additionally, feeding bioessays showed that the sarcotesta extracts of *M. perezfarrerae* and *M pugana*, species endemic to Mexico, were 95% and 93% effective, respectively, against *Anastrepha ludens* adults, while the sarcotesta extracts of *M. vovidesii*, were 92% effective against *A. obliqua*, the West Indian fly [49].

## 6. Secondary Metabolites in *Magnolia*

### 6.1. Fruit with Seed

The fruit (polyfollicle) of Magnolias is a structure that is rich in secondary metabolites with specialized metabolic pathways, which are not involved in primary metabolism [50]. The chemical analysis of essential oils derived from fruits of 2 populations of *M. ovata*, a species endemic to Brazil, showed that they possess a wide diversity of metabolites, including 49 volatile constituents, such as α and β-cubebene, butyl heptanoate, and naphtalene, and 14 non-volatile constituents, such as parthenolide, michelenolide, 1-hexadecanol, as well as 3 alkaloids, lysicamine, lanuginosine and O-methylmoschatoline [51].

**Figure 2:**
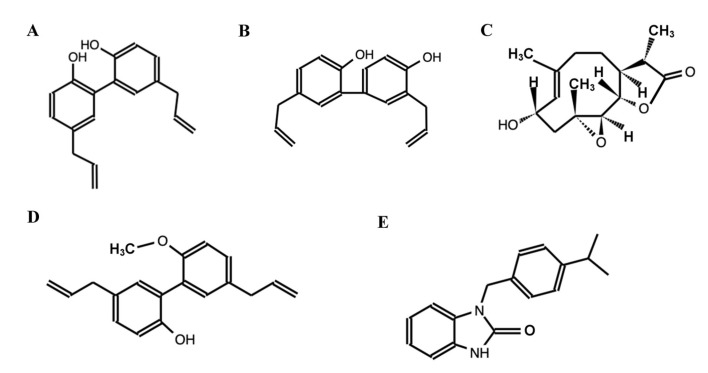
Compounds obtained from *Magnolia grandiflora*. (**A**) 5,5′-diallyl-[1,1′-biphenyl]-2,2′-diol, (**B**) 3′,5-diallyl-[1,1′-biphenyl]-2,4′-diol, (**C**) (3S,3aS,8S,9aS,10aR,10bS,E)-8-hydroxy-3,6,9a-trimethyl-3a,4,5,8,9,9a,10a,10b-octahydrooxireno [2′,3′:9,10]cyclodeca [1,2-b]furan-2(3H)-one, (**D**) 5,5′-diallyl-2′-methoxy-[1,1′-biphenyl]-2-ol, and (**E**) 1-(4-isopropylbenzyl)-1,3-dihydro-2H-benzo[d]imidazol-2-one (Source: [52]).

The phytochemicals contained in the essential oils of ripe fruits with seed of *M. grandiflora* comprised 49 terpenes, such as α-pinene, ethyl 2-methylbutyrate, and isobutyl isobutyrate, and 3 fatty acids, such as (Z)-9-methyl octadecanoate (=methyl oleate), (Z.Z)-9,12-methyl octadecadenoate (=methyl linoleate), and hexadecanoic acid [46]. Additionally, the phytochemical study of immature fruits with seeds of *M. grandiflora* reported the isolation of five chemical compounds (Figure 2) [52]. The presence of nitrile functional groups in the compound in Figure 2E suggest their diversity in terms of biological activity and use in the pharmaceutical industry [52,53]. Additionally, research with phytochemicals of fruits of *M. tripetala*, a species endemic to the United States, lead to the isolation of tripetalin A and B, 4′-methoxymagnaldehyde B, magnaldehyde B, magnoquinone, and magnotriol B [54].

On the other hand, in the essential oil derived from the dry and fresh fruits of *M. kobus*, a species endemic to China, Japan, and the Republic of Korea, 17 chemical compounds were found, including α-thujene, α-pinene, and camphene [55]. Similarly, in the fruits of *M. obovata*, a species endemic to Japan and the Republic of Korea, 20 neolignans (including obovatalignan A, magnolol, and honokiol), six phenylethanoid glycosides (such as (1→2)-β-D-allopyranoside, magnoloside D, and magnoloside A), and five phenylpropanoids (including obovatoside A, syringin, and pavonisol) were identified [56,57,58,59,60,61]. Finally, in the fruit of *M. officinalis* var. *biloba,* nine phenylethanoid glycosides, including magnoloside Ia and crassifolioside, were found [62].

### 6.2. Seedless Fruit (Peel)

As with all fruit structures of *Magnolia* species, the seedless fruit (or fruit follicles) contain a wide variety of natural compounds. In a recent study, the phytochemicals contained in the essential oils of ripe seedless fruits of *M. grandiflora* were analyzed [46]. A total of 43 compounds were identified in this structure, including α-pinene, 1,8-cineole, *p*-cymene, terpinolene, bornyl acetate, α-humulene, myrtenol, and T-cadinol. From the analysis of essential oils from seedless fruits of the species *M. acuminata*, *M. grandiflora*, *M. fraseri,* and *M. tripetala*, species endemic to Canada and the United States, 34 volatile compounds were identified, including α-pinene, β-myrcene, limonene, eucalyptol, borneol, and trans-nerolidol [63]. Likewise, in the seedless fruits of *M. vovidesii*, 15 compounds were isolated from sesquiterpene lactones, such as shizukolidol, and phenols, such as protocatechuic acid, among others [64]. In another study, from the essential oil derived from the peel of *M. kwangsiensis*, a species endemic to China, 21 volatile compounds (including cis-4-thujanol, borneol, and guaiol) were obtained, as well as 10 fatty acids (including heptadecanoic acid, linoleic acid, and heneicosanoic acid [39].

### 6.3. Seed

**Figure 3:**
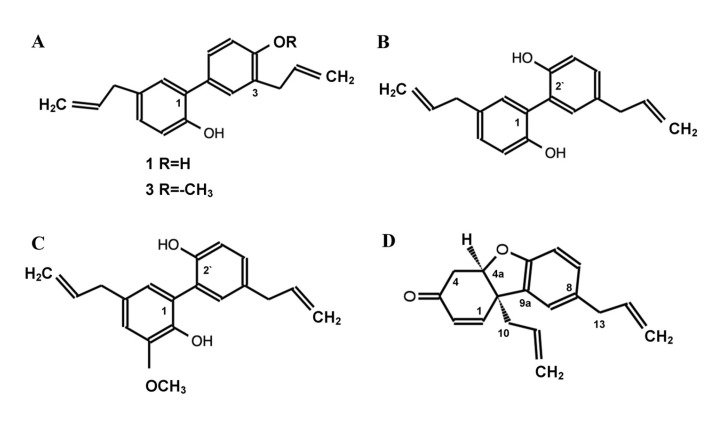
Phenylpropanoids from the seeds of *Magnolia grandiflora*. (**A**) **1**—Honokiol. (**A**) **3**—4-O-metil-honokiol. (**B**) Magnolol, (**C**) 5,5′-di-2-propenyl-3-methoxy-[1,1′-biphenyl]-2,2′-diol. (**D**) 4a,9b-dihydro-8,9b-di-2-propenyl-(4H)-dibenzofuran-3-one, grandifloralignan (Source: [65]).

*Magnolia* seeds also contain a wealth of natural chemical compounds. Via the purpose of isolating lignans, extracts of dichloromethane from seeds of *M. grandiflora, M. acuminata,* and *M. virginiana* (species endemic to Cuba and the United States) were analyzed, including five phenylpropanoids from *M. grandiflora* (Figure 3), honokiol, and magnolol from *M. virginiana*, galgravin, and veraguensin from *M. acuminata* [65]. Another analysis of the compounds in the essential oil of *M. grandiflora* seeds reported the presence of 14 chemical compounds, such as 4-(2-propenyl)-phenol, tetradecanoic acid, eucalyptol, and 2,3-dihydroxy-anti-oleic acid ester [66]. The methanolic extract of *M. grandiflora* seeds presented the neolignans, honokiol and bishonokiol [67]. In a seed hydrodistillate of *M. denudata*, 17 chemical compounds were found, including *p*-cymene, 𝛽-caryophyllene, nerolidol, and ethyl palmitate [47]. The CG-MS chemical analysis of the essential oil from seeds of *M. pugana* obtained 33 chemical compounds, such as isovalerate isobutyl, α-bergamotene, germacrene D, cyclocolorenone, and dehydrosaussurea lactone [68].

### 6.4. Sarcotesta (Aril)

Since the majority of *Magnolia* seeds studies are conducted using whole seeds with sarcotesta, only a small amount is known about the secondary metabolites’ profile of this particular structure. The chemical analysis of an essential oil from the sarcotesta of *M. kwangsiensis* produced 21 terpenes, among them *p*-menth-2-ene, β-phellandrene, acoradiene, and guaiol, and 10 fatty acids, including pentadecanoic, linoleic, eicosanoic, and heneicosanoic, were among them [39].

**Figure 4:**
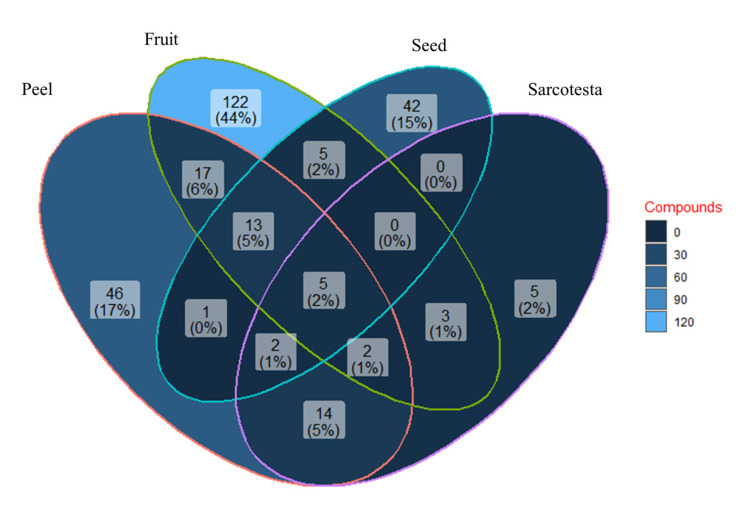
Venn diagram with the distribution of 277 chemical compounds in each of the plant structures of 11 *Magnolia* species, represented by the following colors: fruit (green), peel (red), seed (blue), and sarcotesta (purple), as well as intersection percentages. The compounds were obtained from *M. acuminata*, *M. fraseri*, *M. grandiflora*, *M. kobus*, *M. kwangsiensies*, *M. obovata*, *M. ovata*, *M. pugana*, *M. tripetala*, *M. virginiana*, and *M. vovidesii*.

A search conducted of the scientific literature obtained 277 chemical compounds in the *Magnolia* plant structures, attesting to the wide variety of metabolites contained in each of them. The Venn diagram (Figure 4) shows that fruit with seeds is the plant structure with the largest number of exclusive chemical compounds (122), followed by seedless fruit or peel (46), seed (42), and sarcotesta (5). Additionally, fruit with seeds was the structure that presented the largest number of chemical compounds shared with other structures: it shares 17 chemical compounds with fruit without seeds, 13 with the latter one and with seeds, and 1 compound it shares fruit without seeds with seeds. Finally, only five chemical compounds are shared by all the structures (Table S1).

A large part of these compounds has been individually evaluated for different plague insect species. Boulogne et al., [69] pointed out that terpenoids, phenolic compounds, and alkaloids are the most frequently reported compounds in relation to protection from insects. These three types of compounds are the main secondary metabolism groups involved in the ecological interactions of plants, such as competition and herbivory [70].

## 7. Terpenoids

Terpenoids is a group of secondary metabolites composed of isoprene molecules, units of five carbons (C5), which are known as isopentenil diphosphate (IPP), and dimetilalil diphosphate (DMPP) [71]. In plants, it is possible to synthesize IPP and DMPP following the route of cytosolic mevalonate derived from acetyl-CoA (MEV) and the route of 2-C-metil-D-eritritol-4-phosphate (MEP) plastidial derived from pivurate [72]. Terpenoids are very heterogeneous substances in structure and property, though the majority of them are fat soluble and can be found mainly in essential oils. They stand out for their various degrees of volatility and the influence they have on community and ecosystem interactions [73].

Monoterpenes (C10) and sesquiterpenes (C15) have high degrees of volatility, and they are referred as “inferior terpenoids” [74]. Plants release them in direct defense after an attack by microorganisms, insects, or mammals, but they also have the indirect defense property of attracting the natural predators of attacking herbivores [75]. C10 and C15 are released in complex blends that confuse herbivores and inhibit their capacity to develop resistance to the substances [73].

For these reasons, terpenoids have proved to have insecticidal and anti-nutrient effects on several species of plague insects. However, only a small percentage of monoterpenoids and sesquiterpenoids (approx. 16%) obtained from *Magnolia* fruits with and without seeds and sarcotesta have been tested on insects and other arthropods (Table 1). This small part can have an important role as bioinsecticides and replace the indiscriminate use of harmful pesticides. Unassayed terpenoids (84%) remain an opportunity for research on bioactivity against several pests [76]. Their synergic effects could be explored by trying them in different combinations. Moreover, the effectiveness of existing natural extracts and essential oils can be enhanced via adding extra terpenoids [77,78].

## 8. Phenols

Phenolic compounds are characterized as having at least one aromatic ring with one or more hydroxyl groups in combination. They are classified as non-flavonoids or flavonoids [98]. Non-flavonoids include free phenols, phenolic acids, and phenylpropanoids, which in turn are divided into hydroxycinnamic acids, coumarins, phenylpropanoids, lignins, lignans, and neolignans. Flavonoids, on the other hand, are classified into more than 10 classes, including flavones, isoflavones, flavonoids, flavanones, stilbenes, and anthocyanins [99]. The synthesis of phenolic compounds in superior plants happens in two ways: (1) via shikimic-phenylpropanoids (predominantly in plants) and/or (2) via malonate-polyketide-phenylpropanoids (predominantly in bacteria, fungi, and plants) [100].

Phenolic compounds have an ecological function, namely, to prevent nutrient loss in plants as a result of the feeding behavior of phytophaga [70]. They act also as protection agents against the effects of abiotic factors (sunlight and low temperatures) that indirectly modify plant growth, mineral nutrition, and pigment and aroma in flowers and fruits, and additionally, they act as natural toxic inhibitors for animals and invasive organisms [101,102].

It is evident that phenols have the capacity to act as molecules against insects and that they can also have an important role in the protection of crops. Table 2 presents the insecticidal potential of those phenolic components that have been evaluated in different plague insects, which represent approximately 18% of the total compounds reported from Magnolia fruit with and without seeds and sarcotesta. It is important to note that a high percentage (82%) of the phenolic compounds obtained have not yet been assayed (Table 2) either in the laboratory or in the field to determine their insecticidal effect on several plagues.

## 9. Alkaloids

Alkaloids are organic substances containing nitrogen, with a high or low degree of base quality [116]. In the case of *Magnolia*, alkaloids of the aporphine type belonging to the class of isoquinolines were identified in the fruit of *M. ovata* [51,52,53,54,55,56,57,58,59,60,61,62,63,64,65,66,67,68,69,70,71,72,73,74,75,76,77,78,79,80,81,82,83,84,85,86,87,88,89,90,91,92,93,94,95,96,97,98,99,100,101,102,103,104,105,106,107,108,109,110,111,112,113,114,115,116,117]. Their biosynthesis is achieved via benzylisoquinolines that give way to (s)-reticulin, an intermediary metabolite key to the formation of aporphines [118]. In general, alkaloids are part of the arsenal of chemical defense against herbivores and pathogens [119]. They can be characterized as repellents, deterrents, anti-nutrients, toxic, allelopathics, or germination inhibitors [120]. However, the study of the insecticidal interaction between lysicamine aporphines, lanuginosine, and O-methylmoschatoline has not been determined. This is an area of opportunity to ask new research questions about unassayed alkaloids and get to learn about their bioactivity against pest insects and other organisms.

## 10. *Magnolia*: Between Bioprospection and Conservation

Existing information on the biology of *Magnolia* species remains scarce and, in many cases, restricted to the study of taxonomic aspects [21,121]. According to the Red List of Magnoliaceae, 85% of species are at risk or have insufficient data [21]. The indiscriminate felling of trees, land use changes, and high fragmentation rates of native habitats form a scenario in which practically the totality of *Magnolia* species are under some degree of threat [122]. Moreover, the susceptibility of *Magnolia* increases as the inadequate conditions of restricted habitats and the predation from soil-bound organisms prevent the successful dispersion and germination of seeds [123].

If we add knowledge about existing lacunae and the physiological and genetic aspects of these plants, the geographical distribution patterns of plant diversity, in general, the effects and responses to anthropic processes, and the mechanisms for conservation to this picture, it becomes evident that developing strategies to help and promote the conservation of *Magnolia* species must be considered as a priority [43,124,125].

The bioprospection of plants for the obtention of bioinsecticides based on natural extracts and secondary metabolites can produce more information about this group of species. This would attract the attention of researchers and elicit interest in their preservation via correct methods, tactics, and planning to ensure the equilibrium and dynamics of *Magnolia* populations [126]. In the agricultural sector, *Magnolia* natural products have the potential to be part of integrated pest control management and contribute to a sustainable agriculture free from the use of dangerous synthetic pesticides [127].

The following question arises then: how can *Magnolia* be used to protect crops? Judging from the data obtained in the revision of scientific literature, we believe that the ideal sources of bioprospection are the sarcotesta and seedless fruit. When they are not consumed by a dispersal agent, the sarcotesta inhibits the seed germination process, and the fruit, once empty, decomposes into organic matter, a viable source of bioactive components [128,129]. Removing both seeds and sarcotesta improves the germination process and contributes to the in situ and ex situ propagation of species [43]. The implementation of a restoration strategy via reinstating individuals to their natural populations (in situ conservation) can be complemented by ex situ conservation in botanical gardens, natural protected areas, and germplasm banks [21]. These actions combined will help to reduce the risk of extinction. Finally, it has been observed that seeds are a great source of secondary metabolites, and so, it is important to isolate the existing bioactive insecticides and evaluate their effectiveness in pest control around the world.

Currently, Honokiol and Magnolol isolated from *Magnolia officinalis* are available on the active compounds market to treat several human diseases, respiratory diseases [22,130], cancer [131,132], obesity [133], intestinal problems, and gastric disturbances [22]. In this review, we suggest that the use of *Magnolia* active compounds against agriculture pests has a huge economic potential, for example: (I) Terpenoids: β-caryophyllene has an insecticide effect on Hymenoptera and Coleoptera [80,87], β-phellandrene and α-terpinene have an insecticide effect on Coleoptera [83,84], *p*-cymene has an insecticide effect on Diptera [85], and E-nerolidol has an insecticide effect on Hemiptera [93]. (II) Phenols: 2,4-di-tert-butylphenol has an insecticide effect on Trombidiformes [103], Protochatecuic acid has an insecticide effect on Diptera [110], Rutin has an insecticide effect on Orthoptera [113], and Estragol and Quercetin have an insecticide effect on Lepidoptera [104,111,112].

## 11. Conclusions

The interest in the potential of botanical pesticides in agricultural practices to reduce the use of synthetic ones is on the rise. *Magnolia* species are an ideal source of natural bioactive insecticides. As shown in previous studies, seedless fruit and sarcotesta constitute an excellent source for the study of phytochemicals with potential pest control properties. In this study, we considered these two plant structures and their possible role in the production of natural crop-protection ingredients. However, *Magnolia* species are vulnerable, and the extraction of any part of the plant for research on natural products and the obtention of raw extracts must follow a strict procedure and be managed according to local demographic conditions.

## Figures and Tables

**Table 1:** Biocidal potential of *Magnolia* terpenoids against insects and other arthropods. Compounds were isolated from *M. acuminata*, *M. fraseri*, *M. grandiflora*, *M. kobus*, *M. kwangsiensies*, *M. obovata*, *M. ovata*, *M. pugana*, *M. tripetala*, *M. virginiana*, and *M. vovidesii*. Stages: (A) adult, (L4) fourth instar larva, (L3) third instar larva, and (L) larva.

Compound	Activity	Species	Orden	Stages	LD50	Time	Exposition	References
α-caryophyllene	Insecticide	*Helicoverpa armigera*	Lepidoptero	L3	20.86 µg/mL	24 h	Intake	[79]
α-humulene	Insecticide	*Dorymyrmex thoracicus*	Hymenoptera	A	75 μL/L	48 h	Spraying	[80]
α-phellandrene	Insecticide	*Sitophilus zemais*	Coleoptera	A	15.61 mg/L	24 h	Spraying	[81]
α-pinene	Insecticide	*Lycoriella mali*	Diptera	A	9.85 µL/L air	24 h	Spraying	[82]
	Insecticide	*Tribolium castaneum*	Coleoptera	A	14.08 mg/L air	24 h	Spraying	[83]
α-terpinene	Insecticide	*T. castaneum*	Coleoptera	A	23.70 μL/L air	24 h	Spraying	[84]
	Insecticide	*Musca domestica*	Diptera	A	2.41 μL/L	24 h	Spraying	[85]
α-terpineol	Larvicide	*Culex pipiens molestus*	Diptera	L4	194 mg/L	24 h	Contact	[81]
	Insecticide	*M. domestica*	Diptera	A	3.74 μL/L	24 h	Spraying	[85]
Ar-curcumene	Larvicide	*An. stephensi*	Diptera	L3	10.45 µg/mL	24 h	Contact	[86]
	Larvicide	*Cx. quinquefasciatus*	Diptera	L3	12.24 µg/mL	24 h	Contact	[86]
β-caryophyllene	Insecticide	*D. thoracicus*	Hymenoptera	A	1.49 μL/L	48 h	Spraying	[80]
	Insecticide	*T. castaneum*	Coleoptera	A	36.0 μg/adult	24 h	Contact	[87]
β-myrcene	Insecticide	*M. domestica*	Diptera	A	4.95 μL/L	24 h	Spraying	[85]
β-phellandrene	Insecticide	*T. castaneum*	Coleoptera	A	22.56 mg/L	24 h	Spraying	[83]
β-pinene	Insecticide	*Lasioderma serricorne*	Coleoptera	A	14.66 mg/L	24 h	Spraying	[83]
	Insecticide	*L. mali*	Diptera	A	11.85 µL/l air	24 h	Spraying	[82]
Bornyl acetate	Insecticide	*Liposcelis bostrychophila*	Psocoptera	A	1.1 mg/L air	24 h	Spraying	[88]
	Insecticide	*M. domestica*	Diptera	A	4.24 μL/L	24 h	Spraying	[85]
β-selinene	Insecticide	*Drosophila melanogaster*	Diptera	A	0.55 µg/adult	3 h	Topical application	[89]
Caryophyllene oxide	Larvicide	*An. anthropophagus*	Diptera	L4	49.46 mg/L	24 h	Contact	[90]
	Insecticide	*T. castaneum*	Coleoptera	A	0.00018 mg/cm3	24 h	Spraying	[91]
δ-cadinene	Larvicide	*Anopheles stephensi*	Diptera	L3	8.23 µg/mL	24 h	Contact	[92]
	Larvicide	*Aedes aegypti*	Diptera	L3	9.03 µg/mL	24 h	Contact	[92]
E-nerolidol	Insecticide	*Metopolophium dirhodum*	Hemiptera	A	3.5 mL/L	48 h	Contact	[93]
γ-terpinene	Insecticide	*Phthorimaea operculella*	Lepidoptera	A	5.98 mg/L air	24 h	Spraying	[94]
Guaiol	Insecticide	*M. domestica*	Diptera	A	16.9 µL/L	48 h	Spraying	[95]
	Larvicide	*Plutella xylostella*	Lepidoptera	L3	8.9 mg/larva	12 h	Contact	[95]
Limonene	Insecticide	*T. castaneum*	Coleoptera	A	6.79 mg/L	24 h	Spraying	[83]
	Insecticide	*M. domestica*	Diptera	A	3.22 μL/L	24 h	Spraying	[85]
Linalool	Insecticide	*Sitophilus zeamais*	Coleoptera	A	10.46 mg/L	24 h	Spraying	[96]
	Larvicide	*Cx. pipiens molestus*	Diptera	L4	193 mg/L	24 h	Contact	[97]
*p*-cymene	Insecticide	*T. castaneum*	Coleoptera	A	27.01 μL/l air	24 h	Contact	[84]
	Insecticide	*M. domestica*	Diptera	A	0.77 μL/L	24 h	Spraying	[85]

**Table 2:** Biocidal potential of *Magnolia* phenols against insects and other arthropods. Compounds were isolated from *M. acuminata*, *M. fraseri*, *M. grandiflora*, *M. kobus*, *M. obovata*, *M. ovata*, *M. tripetala*, *M. virginiana*, and *M. vovidesii*. Stages: (A) adult, (L4) fourth instar larva, (L3) third instar larva, and (L) larva.

Compound	Activity	Species	Orden	Stages	LD50	Time	Exposition	References
2,4-di-tert-butylphenol	Acaricide	*Tetranychus cinnabarinus*	Trombidiformes	A	7.61 µM	24 h	Spraying	[103]
Estragol	Larvicide	*Spodoptera frugiperda*	Lepidoptera	A	0.92 mg mL	24 h	Intake	[104]
	Insecticide	*Sitophilus zeamais*	Coleoptera	A	14.10 mg/L	24 h	Spraying	[87]
Eugenol	Insecticide	*Tribolium castaneum*	Coleoptera	A	1 μg/kg	24 h	Contact	[105]
	Insecticide	*Callosobruchus maculatus*	Coleoptera	A	24.8 μL/L	24 h	Spraying	[106]
Honokiol	Larvicide	*Aedes albopictus*	Diptera	L3	6.13 mg/L	24 h	Contact	[107]
	Larvicide	*Anopheles sinensis*	Diptera	L3	7.37 mg/L	24 h	Contact	[107]
	Insecticide	*Nilaparvata lugens*	Hemiptera	A	0.324 mM	48 h	Topical application	[108]
Licarin A	Larvicide	*S. litura*	Lepidoptera	L	0.20% m/m	7 d	Intake	[109]
Magnolol	Insecticide	*N. lugens*	Hemiptera	A	0.137 mM	48 h	Topical application	[108]
	Larvicide	*Culex pipiens pallens*	Diptera	L3	26 mg/L	24 h	Contact	[107]
Protochatecuic acid	Insecticide	*Ae. aegypti*	Diptera	A	1.25 µg/mg	24 h	Contact	[110]
Quercetin	Larvicide	*S. litura*	Lepidoptera	L4	10.88 ppm	24 h	Intake	[111]
	Larvicide	*Pectinophora gossypiella*	Lepidoptera	L	0.2%	Until pupae maturation	Intake	[112]
Rutin	Insecticide	*Oedaleus asiaticus*	Orthoptera	A	763.7 mg/L	7 days	Intake	[113]
Scopoletin	Larvicide	*Spilarctia obliqua*	Lepidoptera	L4	20.9 μg/g	24 h	Intake	[114]
Syringin	Anti-nutrients	*S. granarius*	Coleoptera	A	134.4 μL/L	5 d	Intake	[115]

## Supplementary Materials

The following supporting information can be downloaded at: https://www.mdpi.com/article/10.3390/molecules28124681/s1, Table S1: Compounds identified in *Magnolia*’s fruit parts.
