# Supplementary material for: The Potential of Magnolia spp. in the Production of Alternative Pest Control Substances
Source: Molecules. 2023 Jun 9;28(12):4681. doi: 10.3390/molecules28124681 (PMC10303668; doi:10.3390/molecules28124681)
Supplement: Supplementary file 1 [file molecules-28-04681-s001.zip › molecules-2432452-supplementary.pdf]

Table S1. Compounds identified on *Magnolia*´s fruit parts (Source: [39,46,47,51,52,54–68]).

	Plant Structure			
**Compound**	**Peel**	**Fruit**	**Seed**	**Sarcotesta (aril)**
(-)-spathulenol	X			
(-)-trans-pino-carvylacetate	X			
(1-2)-β-D-allopyranoside		X		
(3S,3aS,8S,9aS,10aR,10bS,E)-8-hydroxy-3,6,9a-trimethyl-3a,4,5,8,9,9a,10a,10b-octahydrooxireno[2′,3′:9,10]cyclodeca[1,2-b]furan-2(3H)-one		X		
(5β, 7β, 7β)-3, 11, eudesmadien-2-one			X	
(E)-nerolidol	X	X	X	
(E)-α-bisabolene	X	X		
(E)-β-farnesene		X		
(E)-γ-bisabolene			X	
(Z)-2-hydroxy-1-(hydroxymethyl)-9-octadecenoic acid ethyl ester			X	
1-(4-isopropylbenzyl)-1,3-dihydro-2H-benzo[d5,5′-diallyl-2′-methoxy-[1,1′-biphenyl]-2-ol]imidazol-2-one		X		
1-decanol		X		
1-heptanol		X		
1-hexadecanol		X		
1-octadecanol		X		
1-octanol		X		
1-pentadecanol		X		
1-terpinen-4-ol	X			
1,2,3,4-tetrahydro-1,6-dimethyl-4(1-methylethyl)-(1S-cis)- naphthalene			X	
1,6-dimethyl-4-(1-methylethyl)-naphthalene, 4-(1,4,5,7-tetramethyl-pyrrolo[3,4-d]pyridazin-6-yl)-phenylamine			X	
1,8-cineole	X			
13-caryophylleine			X	
13-ethyl-3-hydroxy-(14β)-gona-1,3,5,7,9-pentaen-17-ketone			X	
14-hydroxy-9-epi-E-caryophyllene		X		
14-hydroxy-Z-caryophyllene		X		
14-hydroxy-α-humulene			X	
14-hydroxy-α-muurolene		X		
14-hydroxy-ð-cadinene		X		
2-hydroxyobovaaldehyde		X		
2-isopropyl-5-methyl-Anisole	X			
2-methyl butyl 2-methylbutyrate		X		
2-methylbutyl isovalerate		X		
2-pentadecanone		X		
2,3-dihydroxy-anti-oleic acid ester			X	
2,4-di-tertbutylphenol			X	
3-carene	X			
3′,5-diallyl-[1,1′-biphenyl]-2,4′-diol		X		

4-(2-propenyl)-phenol			X	
4-benzyloxybenzoic acid	X			
4-carene	X			
4-coumaric acid	X			
4-O-demethylkadsurenim M		X		
4-O-metil-honokiol			X	
4-pydroxybenzoic acid	X			
4′-methoxymagnaldehyde B		X		
5,5′-Di-2-propenyl-3-methoxy-[1,1′-biphenyl]-2,2′-diol			X	
5,5′-diallyl-[1,1′-biphenyl]-2,2′-diol		X		
5,5′-diallyl-2′-methoxy-[1,1′-biphenyl]-2-ol		X		
6,9,12,15-docosatetraenoic acid methyl ester			X	
7-epi-virolin		X		
8-cedren-8-ol			X	
9-methoxyobovatol		X		
9-oxofarnesol	X			
acoradiene				X
acuminatin		X		
allo-aromadendrene epoxide			X	
amorpha-4,9-dien-14-al		X		
amorpha-4,9-dien-2-ol		X		
androsta-1,4,6-triene-3,17-diketone			X	
ar-curcumene		X		
aristolone		X		
aromadendrene oxide	X			
artemesia alcohol				X
astragali	X			
behenic acid			X	
benzyl benzoate	X			
benzyl hexanoate	X			X
bicyclogermacrene		X		
borneol	X			
bornyl acetate	X	X		
butyl heptanoate		X		
butyl-2-methylbutyrate		X		
cadalene		X		
cadina-1,4-diene		X		
calamenene	X	X		
camphene	X	X		
camphor	X			
carissone		X		
caryophylladienol I	X			
caryophyllene oxide	X	X		
caryophyllenol I	X			
chavicol		X		
chlorogenic acid	X			
cis-11-eicosenoic acid	X			X
cis-4-thujanol	X			X
cis-nerolidol			X	
cis-β-farnesene				X

cis-β-ocimene	X			X
copaene				X
costunolide	X			
crassifolioside		X		
cyclocolorenone		X	X	
dehydrosaussurea lactone			X	
E-caryophyllene		X	X	
E-nerolidyl acetate		X		
eicosanoic acid	X			X
ent-espatulenol			X	
ethyl 2-methylbutyrate		X		
ethyl hexadecanoate		X		
ethyl palmitate			X	
eucalyptol	X	X	X	
eudesm-7(11)-en-4-ol			X	
eudesma-4(14),11- diene	X			
eugenol		X		
farnesene	X			
fenchone		X		
galgravin			X	
geranic acid			X	
geranyl acetone		X		
germacrene B		X	X	
germacrene D	X	X	X	
germacrene-D-4-ol			X	
globulol	X	X		
grandifloralignan			X	
guaia-6,10(14)-dien-4β-ol	X			
guaiol	X	X		X
helifolenol A			X	
heneicosanoic acid	X			X
heptadecane		X		
heptadecanoic acid	X			X
hesperidin	X			
hexadecanoic acid	X	X	X	X
hexadecenoic acid	X			X
honokiol		X	X	
humulene epoxide-II	X	X		
isobiciclogermacrenal			X	
isobicyclogermacrenal		X		
isobutyl 2-methylbutyrate		X		
isobutyl 3-methylbutyrate		X		
isobutyl isobutyrate		X		
isoledene		X		X
isomagnolol		X		
isoobovatol		X		
isovalerate isobutyl			X	
kadsurenin M		X		
khusilal		X		
khusinol acetate		X		
lanuginosine		X		
licarin A		X		
limonene	X	X	X	

linalool	X	X		
linoleic acid	X	X	X	X
longipinocarvona			X	
lysicamine		X		
magnaldehyde B		X		
magnobovatol		X		
magnolol		X	X	
magnoloside A		X		
magnoloside F		X		
magnoloside G		X		
magnoloside H		X		
magnoloside Ia		X		
magnoloside Ib		X		
magnoloside Ic		X		
magnoloside IIa		X		
magnoloside IIb		X		
magnoloside IIIa		X		
magnoloside IVa		X		
magnoloside V		X		
magnoquinone		X		
magnotriol B		X		
magnovatin A		X		
magnovatin B		X		
methyl caffeate		X		
methyl chavicol		X		
methyl hexadecanoate		X		
methyl linoleate		X		
methyl linolelaidate			X	
methyl oleate		X		
mexicanin	X			
michelenolide		X		
mustakone		X		
myrcene		X		
myrtenal	X			
myrtenol	X			
N-(6-Oxo-9,10,11,12-tetrahydro-6H-5-oxa-8-aza-benzo[C] phenanthren-7-yl)-propionamide			X	
n-hexadecane			X	
naphtalene		X		
neryl propanoate		X		
nerolidol			X	
O-methylmoschatoline		X		
obovaaldehyde		X		
obovatal		X		
obovatalignan A		X		
obovatalignan B		X		
obovatalignan C		X		
obovatalignan D		X		
obovatalignan E		X		
obovatalignan F		X		
obovatalignan G		X		
obovatalignan H		X		

obovatalignan I		X		
obovatol		X		
obovatoside A		X		
obovatoside B		X		
obovatoside C		X		
octadecanoic acid	X			X
octadecenoic acid	X			X
oleiferin A		X		
oleiferin C		X		
p-cymen-8-ol	X			
p-cymene	X	X	X	
p-menth-1-ene	X			
p-menth-2-ene				X
palmitic acid			X	
parthenolide	X	X		
pavonisol		X		
pentadecanoic acid	X			X
perillyl alcohol	X			
perillene		X		
pinocarvone	X			
propyl 2-methylbutyrate		X		
protocatechuic acid	X			
quercetin	X			
rutin	X			
salvial-4(14)-en-1-one		X		
scopoletin	X			
scopolin	X			
selin-11-en-4α-ol	X			
sesquisabinene		X		
spathulenol		X		
syringin		X		
terpenene-4-ol	X			
terpinen-4-ol	X	X	X	
terpinolene	X			X
tetradecanoic acid			X	
trans-nerolidol	X		X	
trans-pinocarveol	X			
trans-pinocarvyl acetate	X			
trans-β-bergamotene	X	X		
trans-β-guaiene		X		
tripetalin A		X		
tripetalin B		X		
vanillic acid	X			
vanillin	X			
veraguensin			X	
verbenone	X			
α-acorenol		X		
α-bergamotene			X	
α-cadinol	X	X	X	
α-calacorene	X	X		
α-caryophyllene			X	
α-copaene	X	X		
α-cubebene		X		

α-elemene	X			
α-eudesmol		X		
α-gurjunene			X	
α-humulene	X	X	X	
α-muurolene	X	X		
α-myrcene	X			
α-phellandrene	X	X		X
α-pinene	X	X	X	X
α-selinene	X	X	X	
α-terpinene	X		X	X
α-terpineol	X	X	X	X
α-thujene		X		
α-trans-bergamotene		X		
α-ylangene		X		
α, *p*-dimethylstyrene	X			
β-bisabolene		X		X
β-caryophyllene	X	X	X	
β-cedren-9-one			X	
β-cubebene		X		
β-elemene	X	X	X	
β-eudesmol		X		
β-linalool	X			X
β-myrcene	X			X
β-phellandrene		X		X
β-pinene	X	X	X	
β-selinene	X	X		
γ-cadinene	X	X		
γ-elemene		X		
γ-muurolene	X	X		
γ-terpinene	X		X	X
δ-cadinene	X	X	X	X
τ-cadinol	X	X		
τ -muurolol	X	X	X	
